# Magnetic resonance imaging indicator of the causes of optic neuropathy in IgG4-related ophthalmic disease

**DOI:** 10.1186/s12880-019-0347-z

**Published:** 2019-06-18

**Authors:** Jing Li, Yan Zhang, Hang Zhou, Lei Wang, Zhenchang Wang, Hongyang Li

**Affiliations:** 1 Department of Ophthalmology, Beijing Friendship Hospital, Capital Medical University, No. 95 Yong’an Road, Xicheng District, Beijing, 100050 People’s Republic of China; 2 Department of Radiology, Beijing Friendship Hospital, Capital Medical University, Beijing, China; 3 Department of Ophthalmology, PLA Army General Hospital, No.5, nanmencang, Dongsishitiao, dongcheng district, Beijing, 100000 China; 4 Department of Rheumatology, Beijing Friendship Hospital, Capital Medical University, Beijing, China; 5 Department of Ophthalmology, PLA General Hospital, No.28, fuxing road, haidian district, Beijing, China 100080

**Keywords:** IgG4-related ophthalmic disease, Optic neuropathy, Magnetic resonance imaging, Cause of disease

## Abstract

**Background:**

The following study investigates the involvement of optic neuropathy in IgG4-related ophthalmic diseases (IgG4-ROD) based on the magnetic resonance imaging (MRI) data, and different imaging features of IgG4-ROD related optic neuropathy related to other orbital diseases.

**Methods:**

This retrospective study included 225 patients with IgG4-RD admitted at two ophthalmology centers between January 2014 and December 2017. Twenty-six patients had both pre-therapeutic orbital MRI and optic never injury. The causes of optic neuropathy were analyzed, and the special sign in MRI to diagnose IgG4-ROD was also evaluated.

**Results:**

Twelve cases had inflammation of the optic nerve sheath, while 14 cases had compression due to extraocular muscles and pseudo tumor masses. Two cases had hypertrophic cranial pachymeningitis, while one case had hypophysis involving optic chiasma.

**Conclusion:**

The most common causes of optic nerve injury in IgG-4 ROD are inflammation of optic nerve sheath, compression of extraocular muscles, pseudo tumor mass and hypertrophic cranial pachymeningitis, and hypophysis involving optic chiasma.

## Background

IgG4-related disease (IgG4-RD) is a chronic inflammatory disorder characterized by IgG4-positive lymphoplasmacytic infiltrative lesions and elevated serum IgG4 levels [1, 2]. Target organs that are most commonly affected by IgG4-RD are the orbits, salivary and lacrimal glands, lungs, kidneys, prostate, aorta and retroperitoneum, lymph nodes, biliary tree and as visceral inflammatory pseudo tumours. In the USA, 23% of all of IgG4-RD cases, the disease manifests in the eye or orbit. Furthermore, lacrimal gland swelling was found in 4 to 34% IgG4-RD cases in Japanese reviews.

Eyes are often the first most frequent site associated with IgG4-RD [3]. A number of orbital inflammatory cases, formerly considered as other idiopathic lesions, are now regarded as IgG4-related orbital diseases (IgG4-ROD) [4]. Radiology image is important to distinguish IgG4-ROD from other orbital diseases. IgG4-ROD usually presents as dacryoadenitis, myositis, or inflammation of infraorbital fat. In addition, some researches have indicated that the presence of infraorbital nerve enlargement (IONE) is considered as a key sign of IgG4-ROD on an MRI [5].

Optic neuropathy may be involved in IgG4-ROD, and it can lead to severe injuries. However, this type of disease is more likely misdiagnosed as other orbital inflammatory diseases, such as optic neuritis, Graves’ disease, inflammatory pseudotumor, lymphoma, sarcoidosis and so on [6]. IgG4-ROD is quite different from these diseases with reference to treatment and prognosis [3]. Therefore, its early diagnosis is very important. Over the recent years, much knowledge has been gained about the clinical manifestations of IgG4-ROD [7]; yet, most of studies were small case series, and only few explored the imaging manifestations of IgG4-ROD related optic neuropathy. Clinical data have suggested that decreased visual acuity and defective visual field are the first symptoms to appear. Nevertheless, most of the existing studies have focused on dacryoadenitis, myositis and branch of trigeminal neuropathy [8, 9]. In addition, there is insufficient information on radiological features of optic neuropathy with IgG4-ROD, and causes of optic neuropathy in IgG4-ROD.

Hence, the purpose of this study was to investigate the radiological features of optic neuropathy from IgG4-ROD in Chinese patients. Furthermore, we suggest a radiological diagnostic criterion for optic neuropathy cases diagnosed as IgG4-ROD.

## Materials and methods

### Patients

This retrospective study included 225 patients with IgG4-RD admitted at two ophthalmology centers between January 2014 and December 2017. Ocular manifestations were the first symptoms of IgG4-RD. IgG4-RD was diagnosed based on the Japanese Comprehensive Diagnostic Criteria for IgG4-RD [10]: (1) clinical examination showing characteristic diffuse/localized swelling or masses in single or multiple organs; (2) hematological examination showing elevated serum IgG4 concentrations (≥135 mg/dL); (3) histopathological examination showing marked lymphocyte and plasma cell infiltration and fibrosis and Infiltration of IgG4+ plasma cells: ratio of IgG4+ to IgG+ cells > 40% and > 10 IgG4+ plasma cells/high power field (HPF). Based on these criteria, the patients were diagnosed with IgG4-RD (definite: (1) + (2) + (3); probable: (1) + (3); possible: (1) + (2)).

The exclusion criteria were: ocular manifestations were not the first symptoms; pretherapeutic MRI was insufficient for an adequate interpretation; absence of IgG4 testing during the pathology examination; thyroid-related eye diseases and lymphoma and other orbital- related diseases; ocular exam that was insufficient for analysis; patients without visual loss (best corrected visual acuity< 1.0 or visual field defect) or optic neuropathy (optic atrophy or papilledema).

**Fig. 1:**
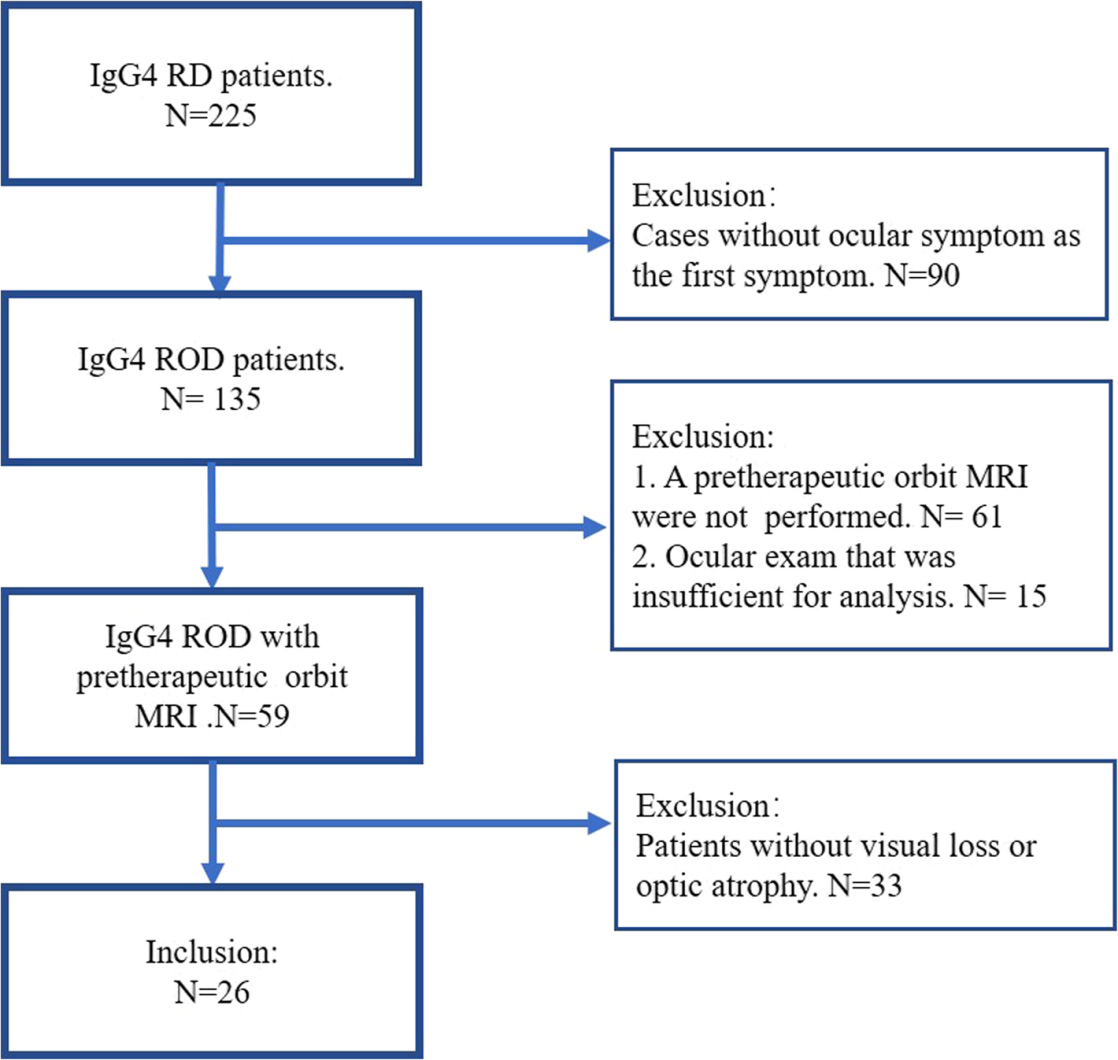
A total of 26 patients met the inclusion criteria

A flowchart illustrating the selection of patients is shown in Fig. 1. A total of 199 patients did not meet the inclusion criteria. Finally, 26 patients were included in the study.

### Review of optic examination

Ophthalmic examinations included slit lamp examination and pupillary reactions in unilateral or bilateral. Visual acuity was examined by the standard table of vision logarithms at 5 m. Those unable to read any letters at the distance of 1 meter were further examined by finger counts, hand movements, or perceiving light. Visual field was performed by Humphrey field Analyzer (30–2 SITA, Humphrey 750i, Zeiss, Germany). Digital fundus photographs were obtained using nonmydriatic fundus photography. Binocular digital photographs were taken using a 45° high-resolution fundus camera (Kowa, Tokyo, Japan); and retinal fundus images were centered on the optic disc.

### Review of MR imaging

The MRI of the orbit was evaluated using MRI-3.0 T (TW1WSPEED HDXT, GE, USA). Scanning sequences and parameters were the following: coronal T1-weighted Fast spin-echo (TR = 660 ms; TE =11.1 ms, matrix size = 256x256mm, FOV = 18x18cm, slice thickness = 3.0 mm); Gd-DTPA 0.1 mmol/kg was used as contrast agent for enhanced MRI, and was combined with fat suppression scan technique. The MR imaging of patients was evaluated by two experienced neuroradiologists (12 and 8 years of imaging experience). From the coronal imaging plane, each extraocular muscle was measured in 2 dimensions (millimeters): maximum diameter and maximum short axis [11, 12].

## Results

### Patient demographics

A total of 26 patients met the inclusion criteria (Table 1). The male: female ratio was 12:14, and the age ranged from 51 to 78 years. There were 23 patients with extraophthalmic involvement, including parotid gland, submandibular gland, sinusitis, pancreatitis, lymph nodes enlargement, nephrosis, cholangitis and pneumonia. All patients had bilateral involvement.

**Table 1:** Demographic and clinical characteristics

Number of patients (n)	26
Age (years)	51–78
Sex (male:female)	12:14
Bilaterality (n)	26
Extrapohthalmic involvement (n)	21
steroid-sensitive patients (n)	23
Auto-antibody (n)	5
ANA	4
SSA/SSB	0
thyroid autoantibodies	0
RF	1
HLA	0
Others	0
Vision loss (n)	26
Vision field defect (n)	26
optic atrophy (n)	10
papilledema (n)	5

### Clinical features of optic nerve injury

**Fig. 2:**
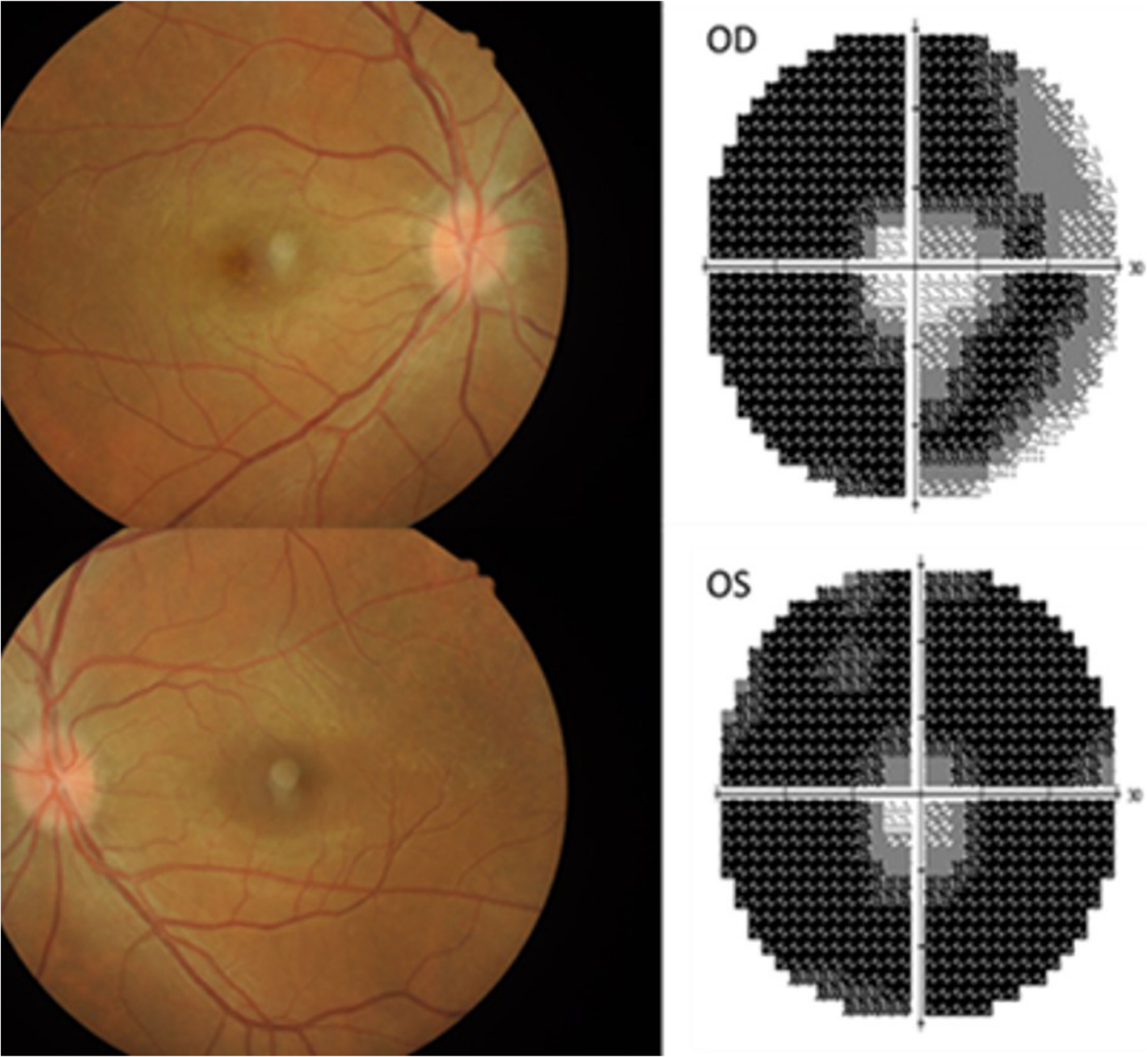
The main performance of optic nerve injury. A patient with bilateral injury from IgG4-ROD. Bilateral papilledema was performed using fundus camera and peripheral visual field defect through Humphrey field Analyzer

The manifestations of optic nerve injury were visual impairment, visual field detection, papilledema or optic atrophy. The most common manifestations of optic nerve sheath inflammation were mild visual impairment, mild papilledema and peripheral visual field defect (Fig. 2).

### MRI indicator of the causes for optic neuropathy in IgG4-ROD

**Fig. 3:**
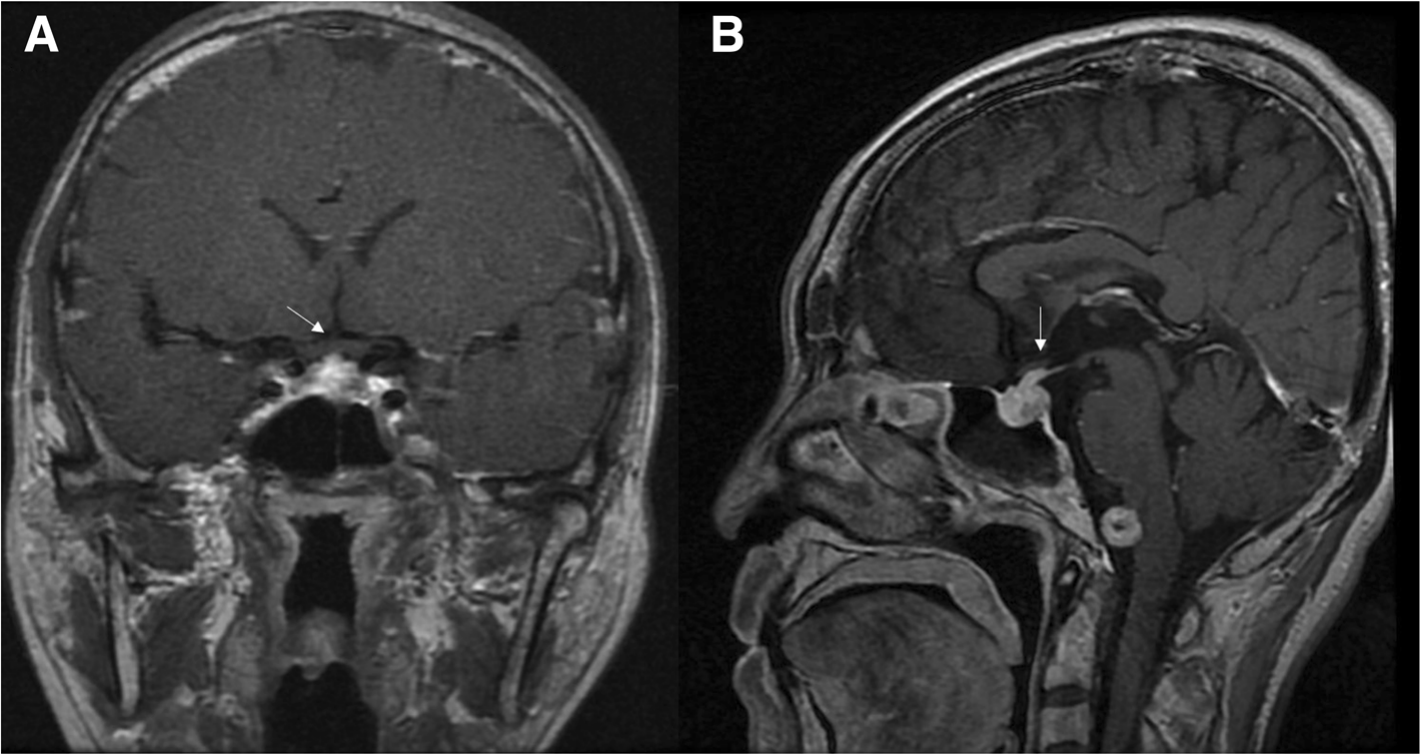
IgG4-RD with hypophysis involving optic chiasma. coronal (**a**) and Sagittal (**b**) contrast enhanced T1-weighted MRI showed diffuse enlargement of the pituitary and infundibulum, resulting in the compression of optic chiasm. White arrows show the opposition of optic chiasm

**Fig. 4:**
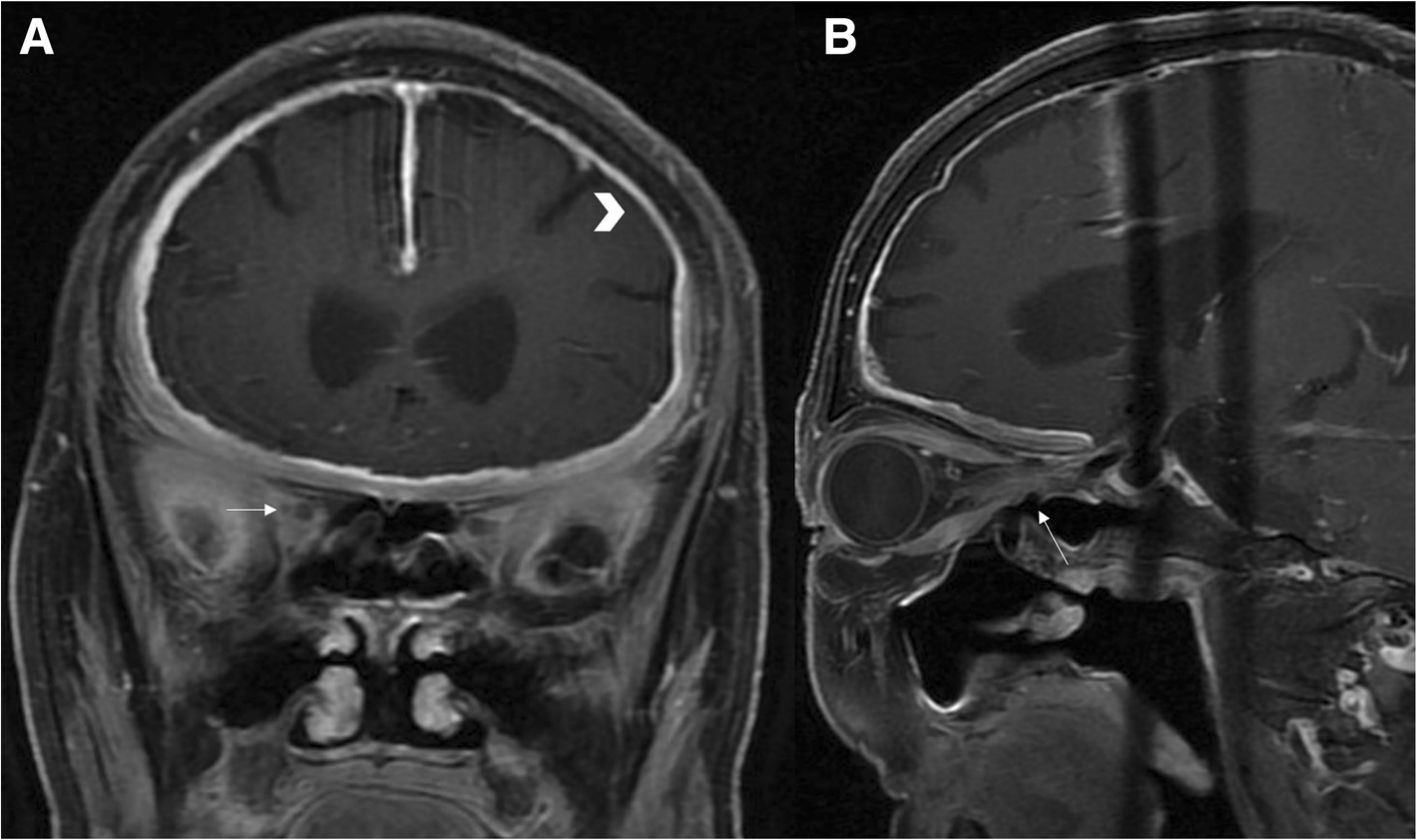
Compression of optic nerve as hypertrophic cranial pachymeningitis. Coronal contrast enhanced T1-weighted MRI (**a**) showing hyperintense linear dura thickening (arrow heads). Sagittal contrast enhanced T1-weighted MRI (**b**) showing the thickened dura mater was markedly enhanced after contrast media, and compressed the optic nerve in the orbit apex (white arrow)

**Fig. 5:**
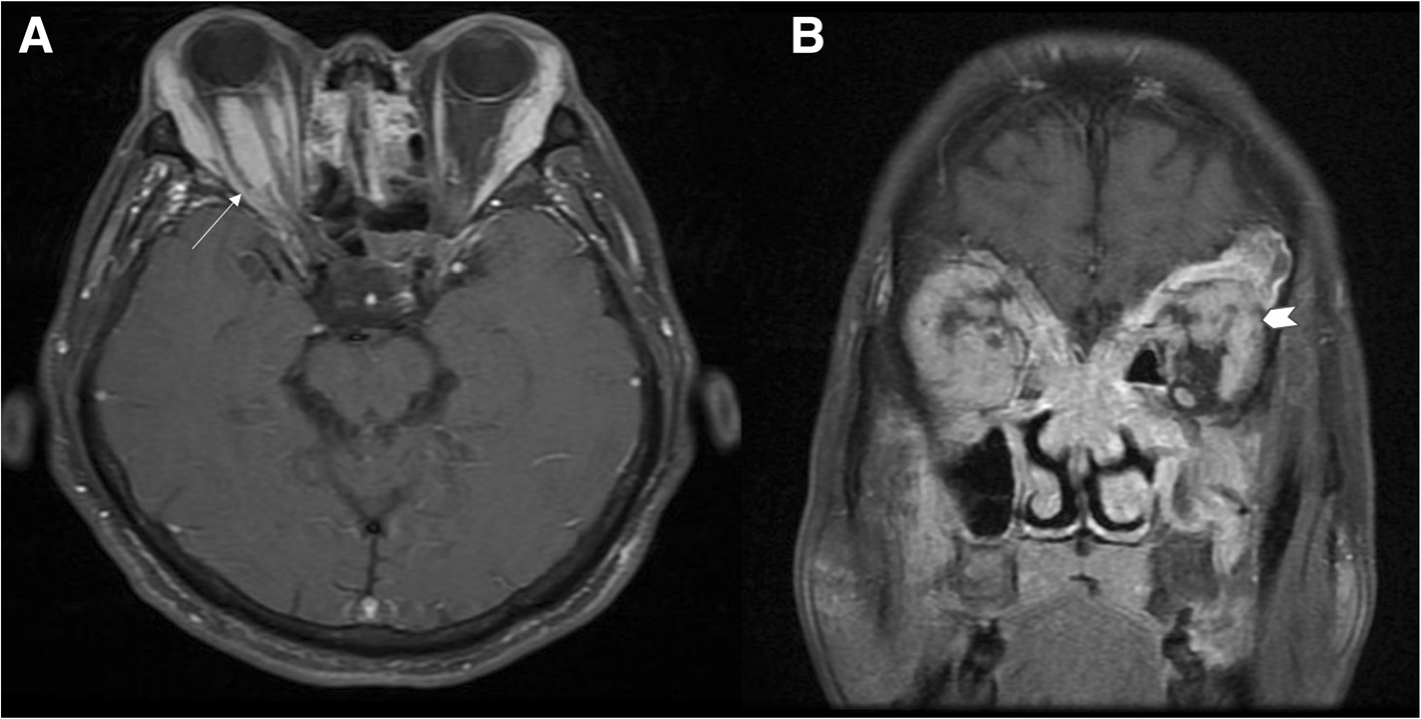
Optic nerve was compressed by pseudotumor masses. Axial and coronal contrast enhanced T1-weighted MRI showing pseudotumor mass circles of optic nerve, was compressed by pseudotumor masses (white arrow) (**a**). Extraocular muscles appeared enlarged as masses and optic nerves were oppressed (white arrowhead) (**b**)

**Fig. 6:**
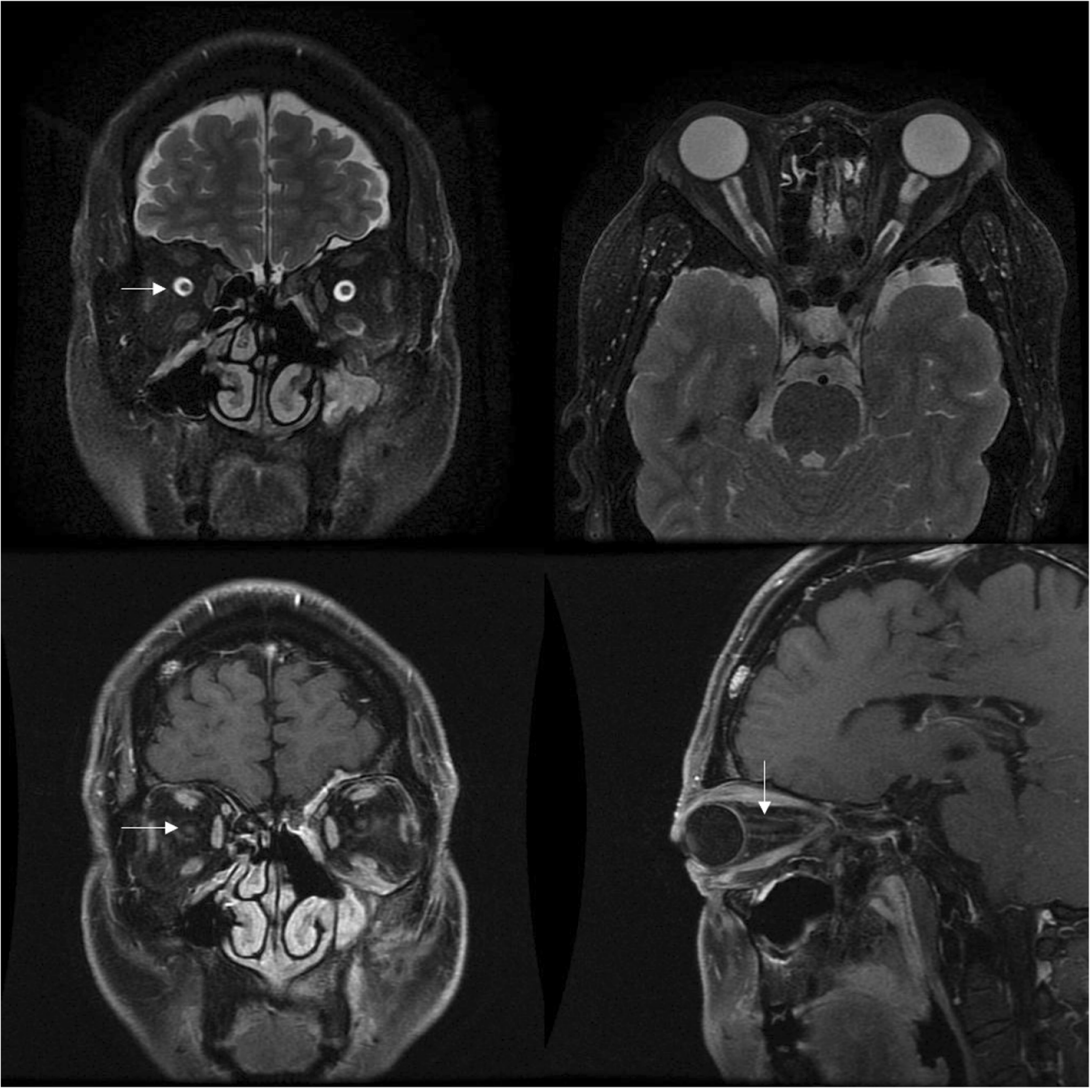
Optic neuropathy with the reason of optic nerve sheath inflammation. Optic neuritis is characterized by diffuse thickening of the optic nerve sheath, blurred margin and enhanced. Optic neuritis was accompanied by orbital liposome inflammation, possibly due to the inflammation of orbital liposome involved in the optic nerve sheath

**Fig. 7:**
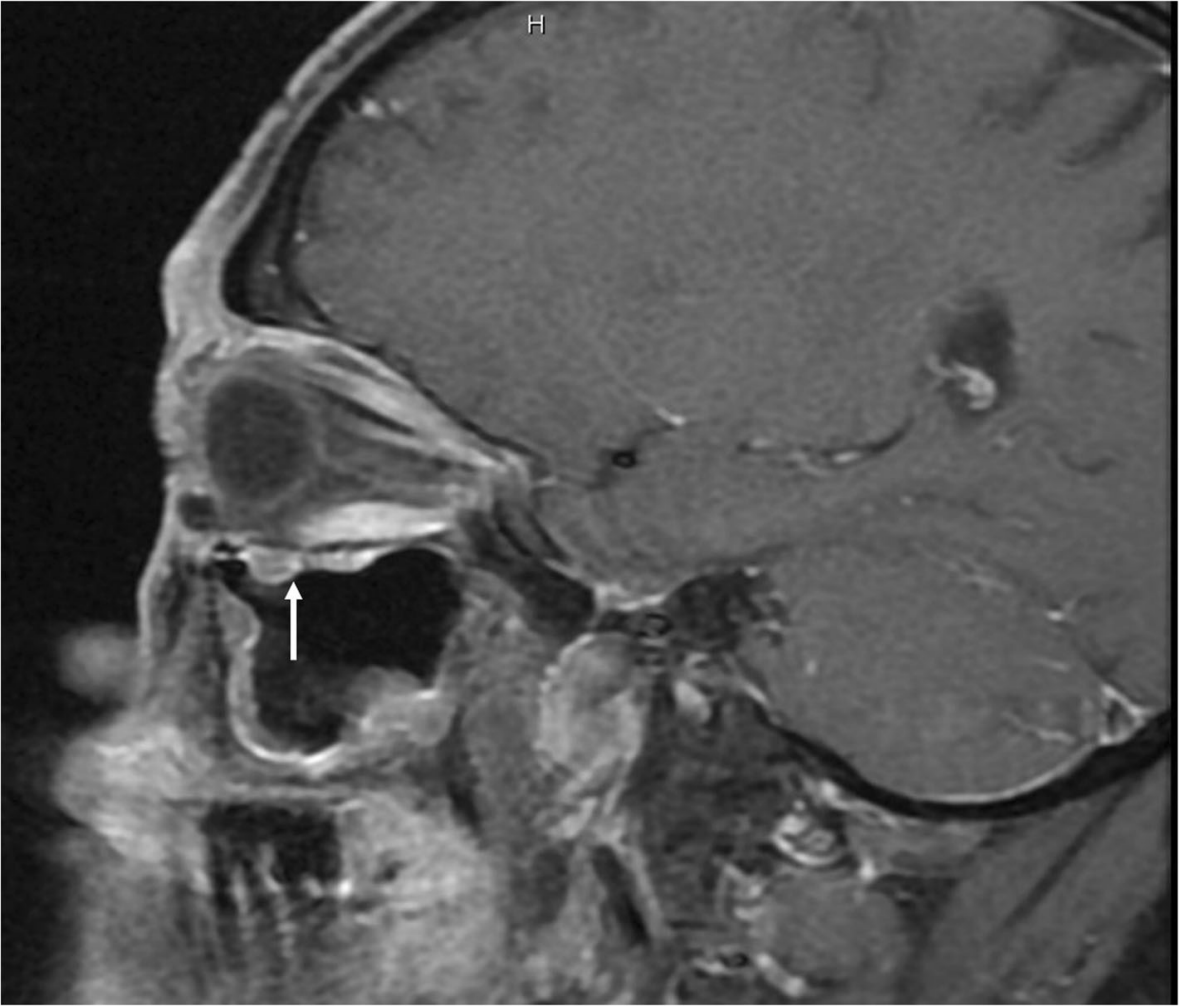
Optic nerve sheath enhancement in patients accompanied with IONE. Sagittal contrast enhanced T1-weighted MRI shows enhancement of optic nerve sheath with IONE (white arrowhead)

Compression was observed in more than half of patients with optic neuropathy, including hypophysis involving the optic chiasm (1 case**,** Fig. 3). MRI showed diffuse enlargement of the pituitary and infundibulum, resulting in the compression of optic chiasm. Two cases with hypertrophic cranial pachymeningitis (Fig. 4), and 14 cases were compressed by pseudotumor masses and extraocular muscles (EOMs) hypertrophy (Fig. 5), MRI showed hyperintense linear dura thickening; thickened dura mater was markedly enhanced after contrast media, compressing the optic nerve in the orbit apex, and pseudotumor mass circles of optic nerve. In 12 patients with optic neuropathy who suffered from optic nerve sheath inflammation (Fig. 6), MRI showed that optic neuritis was enhanced and was characterized by diffuse thickening of the optic nerve sheath, and blurred margin. Furthermore, optic nerve sheath inflammation often appeared with infraorbital nerve enlargement (Fig. 7).

## Discussion

IgG4-RD is characterized by elevated serum IgG4 levels and IgG4-positive lymphoplasmacytic infiltrative lesions [1, 2] and it involves multiple organs, including ocular structures [13, 14]. Previous studies, investigating IgG4-related ophthalmic diseases, have been mainly focused on examining ocular adnexa, dacryoadenitis, myositis and branch of trigeminal neuropathy lesions. In contrast, optic neuropathy is a frequent cause of vision loss encountered by ophthalmologist, which often leads to more severe injuries compered to ocular adnexa lesions. Thus, real-team imaging methods are an important tool for identifying the pathogenesis of optic neuropathy.

In the current study, we used MRI to determine the causes of optic neuropathy development, such as hypophysis, extraocular muscle enlargement, hypertrophic cranial pachymeningitis, compressed by pseudotumor masses and optic nerve sheath inflammation. Currently, the most commonly used imaging modalities for IgG4-ROD are computed tomography (CT) and MRI [15–17]. In the current study, all the patients underwent MRI, since CT imaging of optic nerve and optic nerve sheath were not clear enough. Previous data have indicated that the most common structural lesions include lacrimal gland (62–88% of cases), orbital fat (29–40%), EOM (19–25%), and trigeminal nerve (10–39%) [18, 19]. Other structures may involve eyelid, conjunctiva [20], optic nerve [21, 22] and mass compression. Song et al have found that IgG4-ROD mass lesions possess homogenous internal architecture, well-defined margins, contrast enhancement, and no destruction of orbit bone [23]. In this study, compression was the main cause of optic nerve damage, especially the mass compression, and most EOMs belonged to the pseudotumor mass compression, because the hypertrophy of the EOMs is a characteristic of pseudotumor mass. Previous data have suggested that IgG4-RD has a particular tropism for cranial nerves [24–26]. In addition, it has been reported that IONE can oppress the optic nerve at the orbital apex [27]. However, in this study, we observed no IONE oppression related cases, while most oppressions were caused by pseudo tumor. In the current study, all the cases showed bilateral involvement, while 80.77% (21/26 cases) showed extraophthalmic involvement. Moreover, a Korean study group found that bilateral involvement, longer duration of symptom and higher IgG4 levels are all significant risk factors for extraophthalmic involvement in patients with IgG4-ROD [28]. IgG4-ROD with optic neuropathy involvement was more likely combined with extraophthalmic disorder. Accordingly, optic nerve involvement might be considered as another risk factor for extraophthalmic involvement.

IONE is the main sign for differentiating optic nerve sheath inflammation of IgG4-ROD from other orbital diseases. The orbital region is the first most frequent site of the head and neck region associated with the suffering from IgG4-RD. [3, 4] The main pathogenesis of optic neuropathy is compression; however, optic nerve sheath inflammation is also commonly observed. In addition, optic nerve sheath inflammation is often involved in unidiopathic orbital inflammation diseases [6]. IgG4-ROD diagnoses may be useful for the adequate management and appropriate care of their patients. In the current study, matched patients with non-IgG4 ROD were compared with IgG-4 ROD patients, IONE was found to be a specific sign of IgG4-ROD. A previous research has identified an association between the enlargement of infraorbital branch of the trigeminal nerve and the IgG4-ROD [15, 19, 29]. In previously mentioned study on IgG4-ROD that included European and Japanese study group, IONE was considered as the specific radiological feature [5, 30]. In addition, according to radiological reviews on IgG4-ROD, trigeminal nerve enlargement occured in 24% (4/17 cases) [9], 29% (20/68 cases) [29], 39% (25/65 cases) [19], and 50% (8/16 cases) of cases [15]. In addition, one study (*n* = 14) found that 50% of patients with IONE had IgG4-ROD [26].

Our study has few limitations. This is a study with retrospective design that was conducted only in two centers, thus non-controlled investigation and treatment protocol were considered as limitations. In addition, only 26 cases were included in this study, which means that future studies should include bigger sample size.

## Conclusion

The most common imaging feature of the optic nerve for IgG4 is compression, while inflammation of the optic nerve sheath is the main reason affecting the visual function. Enhancement of optic nerve sheath is very likely to be combined with dacryoadenitis and infraorbital nerve enlargement. In addition, IONE and infraorbital nerve downwards resulted as important indications for the diagnosis of IgG4-ROD.

## Data Availability

All data are contained within the manuscript.

## Abbreviations

EOMs: Extraocular muscles
IgG4-ROD: Immunoglobulin G4 (IgG4)-related ophthalmic diseases
IONE: Infraorbital nerve enlargement
MRI: Magnetic resonance imaging
VA: Visual acuity
